# Cooperative effects of galanin and leptin on alleviation of insulin resistance in adipose tissue of diabetic rats

**DOI:** 10.1111/jcmm.15328

**Published:** 2020-05-12

**Authors:** Bu Le, Xiaoyun Cheng, Shen Qu

**Affiliations:** ^1^ Department of Endocrinology Shanghai 10th People Hospital Tongji University School of Medicine Shanghai China

**Keywords:** adiponectin, Akt, diabetic models, GLUT4, insulin resistance

## Abstract

It was reported that either orexigenic neuropeptide galanin or anorexigenic hormone leptin caught benefit insulin sensitivity through increasing the translocation of glucose transporter 4 (GLUT4) in patients with diabetes. To date, it is unknown whether galanin can potentiate the effect of leptin on alleviation of insulin resistance. Therefore, in the current study we sought to assess the combined effect of central leptin and galanin on insulin resistance in the adipose tissues of type 2 diabetic rats. Galanin and leptin were injected into the intracerebroventricle of the diabetic rats, respectively, or cooperatively once a day for 2 weeks. Then, several indexes of insulin resistance were examined. The results showed that glucose infusion rates in the hyperinsulinaemic‐euglycaemic clamp test, plasma adiponectin content and GLUT4 translocation, as well as Akt phosphorylation in fat cells, were higher, not GLUT4 protein and GLUT4 mRNA expression, but HOMA index was lower in the galanin + leptin group than either one of them. Furthermore, treatment with MK‐2206, an Akt inhibitor, blocked the combined effects of galanin + leptin on alleviation of insulin resistance. These results suggest that galanin can improve the leptin‐induced mitigative effects on insulin resistance in the fat cells, and those provided new insights into the potential tactics for prevention and remedy of insulin resistance.

## 1 INTRODUCTION

The hallmark of insulin resistance is a disturbance in glucose uptake, resulting in hyperinsulinaemia and heperglycaemia. The relation between insulin resistance and subsequent risk of type 2 diabetes is well established. Despite extensive investigations into insulin resistance, the precise mechanism is incompletely understood. A lot of endocrine hormones, including galanin and leptin, are involved in the pathogenesis of insulin resistance for their multiple effects on glucose and lipid metabolism.

Data indicate that galanin can regulate energy homeostasis and insulin sensitivity of animals. Galanin neurons and its three subtype receptors 1‐3 distribute throughout the brain, particularly in the dorsomedial hypothalamus,^1^ where is an important regulative centre of energy metabolism.^2^ An injection of galanin into paraventricular nucleus significantly increased feed intake and bodyweight, as well as circulating non‐esterified fatty acid and lipoprotein lipase levels of the rats.^3^ Animals with metabolic disorder of galanin are easy to develop type 2 diabetes mellitus.^4^ Compared with controls, the galanin knockout mice reduced insulin sensitivity and insulin‐independent glucose elimination, and their food intake,^5^, ^6^ whereas the homozygous galanin transgenic mice exhibited increased metabolic rate of lipid and carbohydrate for improved insulin sensitivity.^7^ The results of our ^8^ and other's studies ^9^, ^10^, ^11^, ^12^ indicated that an intracerebroventricular or abdominal administration of M35, a galanin antagonist, enhanced plasma glucose levels but reduced insulin sensitivity in diabetic rats.

Leptin is a protein synthesized and secreted by adipocytes. Plasma leptin levels in humans and animals are correlated to body fat depot.^13^ Leptin receptors are expressed in the arcuate and ventromedial regions of the hypothalamus, and adipose tissues.^14^, ^15^ Central administration of leptin suppressed bodyweight via decreasing food intake and stimulating energy expenditure of rats.^16^ The leptin transgenic animals showed amelioration of high‐fat‐diet‐induced insulin resistance,^17^, ^18^ whereas the leptin‐deficient ob/ob mice displayed obesity and insulin resistance with hyperinsulinaemia and hyperglycaemia,^19^ which might be reversed by exogenous leptin.^20^

Intriguingly, LepRb, a long‐isoform leptin receptor, is expressed in galanin neurons of brainstem and hypothalamus of mice.^21^ The double‐labelling studies indicated that co‐localization of galanin and leptin receptors distributed in the periventricular nucleus, the arcuate nucleus, the supraoptic nucleus and the lateral hypothalamus.^15^ This co‐localization of galanin and leptin receptors offers an morphologic basis of their interaction. The galanin‐expressing neurons in the periventricular nucleus are a target of leptin to regulate feeding behaviour and energy expenditure,^15^, ^22^ and galanin is an important mediator of leptin action to modulate nutrient reward.^23^ Deletion of galanin gene amplifies leptin‐induced weight loss, suggesting that there is an interaction between galanin and leptin signalling system.^21^ However, the combined effects of central leptin and galanin on insulin resistance have not been identified. Therefore, the current experiment was designed to evaluate the combined anti‐diabetic effects of both hormones in fat cells of type 2 diabetic rats.

## 2 MATERIAL AND METHODS

### 2.1 Reagents

Glucose transporter 4 (GLUT4), Akt and pAkt antibodies were obtained from Santa Cruz Biotechnology Inc; galanin and leptin from Sigma‐Aldrich Inc; 2‐deoxy‐D‐[^3^H]glucose ([^3^H]2‐DG) from Perkin Elmer; Akt inhibitor, MK‐2206, from Selleck Chemicals; and adiponectin and insulin ELISA kits from Uscn Life Science, Inc.

### 2.2 Animal preparation

The experiment was performed on 120 freely moving male Wistar rats weighing 150 ± 10 g. All animals were housed in group on a 12:12‐h light‐dark cycle with ad libitum access to high‐fat food (59% fat, 21% protein and 20% carbohydrate) and water in a climate‐controlled environment (22 ± 2°C at 50%‐60% humidity) for 8 weeks. Then, the rats were treated with 30 mg/kg streptozotocin intraperitoneally.^11^ After another 4 weeks, the animals with over 11.1 mmol/L fasting blood glucose levels were used as diabetic models. All experiments were conducted according to the Guiding Principles for Care and Use of Experimental Animals to minimize the animal suffering, and the experiment was approved by the Tongji University Ethics Committee.

### 2.3 Animal groups

A total of 112 model rats were randomly divided into seven groups of 16 each: diabetic control, galanin group, leptin group, galanin + leptin group, galanin + MK‐2206 group, leptin + MK‐2206 group and both hormones + MK‐2206 group. Besides, 16 rats with normal glucose levels were included in the healthy control group.

### 2.4 Intracerebroventricular injection

All rats were anaesthetized with 50 mg/kg amobarbital sodium (i.p.) and implanted stereotaxically with a guide cannula into the lateral ventricle: anterior‐posterior (AP), −0.8 mm; L, 1.4 mm; and V, 3.3 mm.^11^ The cannula was fixed to the skull by stainless steel screws and dental cement. After recovered from the surgery for 7 d, rats in six agent‐treated groups received an i.c.v. infusion of leptin (0.6 nmol), galanin (0.1 nmol) and MK‐2206 (300 μg/kg), either, respectively, or in combination, once a day for continuous 14 days. Both control groups were intraventricular infusion with the same volume of artificial cerebrospinal fluid.

### 2.5 Hyperinsulinaemic‐euglycaemic clamp and ^3^H‐2DG experiments

Fasted for 12 hours after the last injection, half of the rats in every group (n = 8) were anaesthetized as above and cannulated in the jugular vein for infusion of glucose and insulin and in the carotid artery for sampling.^12^ Insulin was constantly infused in 2 mU/kg·min velocity, and 10% glucose was instilled at variable rates as needed to keep glucose levels at 5 ± 0.5 mmol/L. The glucose injection rate was calculated according to six samples under homeostasis during the experiment.

After fasted for 12 hours, the remaining rats in every group (n = 8) were anaesthetized as above and received an intraperitoneal injection with 250 mg/kg ^3^H‐2DG. At 30 minutes after the treatment, epididymal fat pad and 4 mL artery blood were fast collected and stored at –80°C.

### 2.6 Subcellular fractionation

The washed and minced fat pads were homogenized with homogenization buffer at 4°C.^10^ Then, the homogenate was centrifuged at 13 000 *g* for 20 minutes at 4°C. The ^3^H‐2DG uptake was calculated with part of the supernatant in a liquid scintillation counting (Tri‐Carb 2000, Packard Instrument Co.). The intracellular membranes were obtained by recentrifugation of remaining supernatant at 31 000 *g* for 60 minutes. The pellet after this spin was layered over a sucrose cushion and centrifuged at 75 000 *g* for 60 minutes. The pellet was re‐spun at 39 000 *g* for 20 minutes to yield plasma membranes.

### 2.7 HOMA index

The homeostasis model assessment (HOMA)‐insulin resistance indexes were measured by insulin levels (mU/mL) × blood glucose levels (mmol/L)/22.5. The blood insulin and glucose levels were measured using competitive insulin ELISA kits and glucometer (HMD Biomedical, Taiwan), respectively. All measurements were performed in duplicate, and the mean of two measurements was considered.

### 2.8 Measurement of plasma adiponectin levels

The fast blood adiponectin levels were quantified using competitive ELISA kits in accordance with the manufacturer's directions.

### 2.9 Total RNA extraction and real‐time PCR

Total RNA was prepared using TRIzol reagent from the adipose tissue.^10^ After determination of RNA concentrations by measuring the absorbance at 260‐280 nm, 4 μL RNA as template was reversely transcribed to cDNA by using Revert Aid First‐Strand cDNA Synthesis Kit. Real‐time PCR was performed on an Exicycler™ 96 PCR Machine (LG Company, Korea). Specific primers were designed as follows: GLUT4 5′‐ACAGGGCAAGGATGGTAGA‐3′ and 5′‐TGGAGGGGAACAAGAAAGT‐3′; β‐actin 5′‐GGCTGTGTTGTCCCTGTATG‐3′ and 5′‐AATGTCACGCACGATTTCC‐3′. Real‐time PCR assays were as follows: 95°C × 10 minutes, 40 × (95°C × 30 seconds, 60°C × 30 seconds, 72°C × 60 seconds). The △Ct value was expressed by 2^−△△Ct^, and the β‐actin levels were used as internal controls.

### 2.10 Western blotting

The 50 mg protein samples from the subcellular fractions were separated with a 12% SDS‐PAGE and transferred to a nitrocellulose membrane.^11^ The membranes were then probed with a primary antibody against GLUT4, Akt and pAkt, respectively, and a HRP‐conjugated secondary antibody successively. The signals were detected with chemiluminescence and quantified by densitometry with a HPIAS‐2000 Image Analysis System.

### 2.11 Statistical analysis

All data were expressed as mean ± SEM. Differences between groups were determined via the two‐way analysis of variance, followed by the Tukey's test. A *P *value <.05 was statistically considered significant.

## 3 RESULTS

### 3.1 Bodyweight and food intake

**FIGURE 1:**
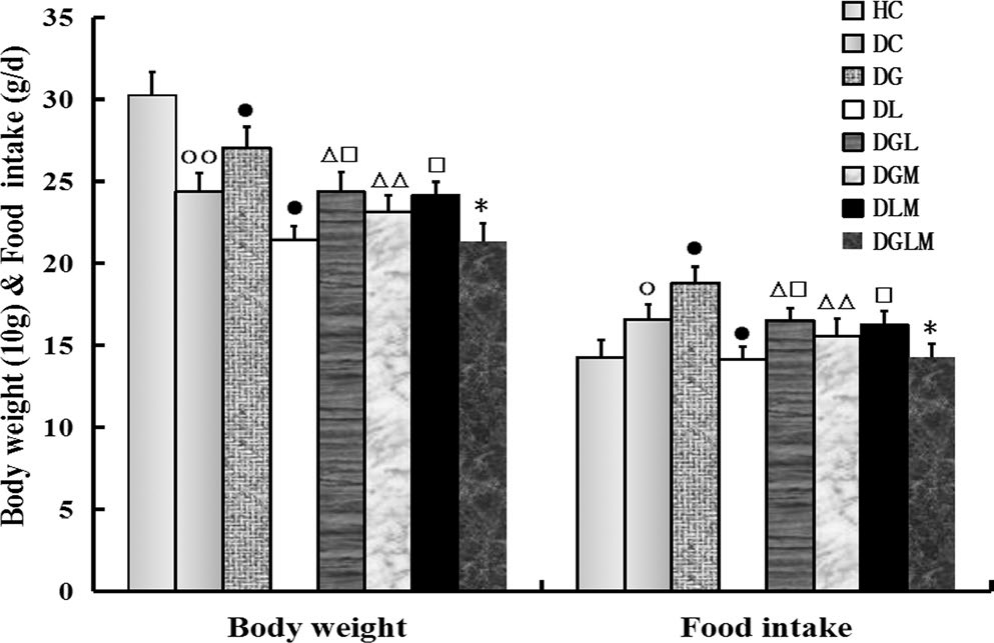
The central effect of galanin and leptin on bodyweight and food intake of rats (n = 8). The bodyweight and food intake of animals were lower in the galanin + leptin group (DGL) or the galanin + MK‐2206 group (DGM) than the galanin group (DG), the both hormones + MK‐2206 group (DGLM) than DGL, and the leptin group (DL) than diabetic controls (DC). But both indexes were higher in DGL or the galanin + MK‐2206 group (DLM) than in DL, in DG than DC. The bodyweight was lower and food intake was higher in DC than the healthy controls (HC). Difference in the both indexes was non‐significant between DGLM and DGM, and between DGLM and DLM. The data are shown as the means ± SEM. ○*P* < .05, ○○*P* < .01 vs HC; ●*P* < .05 vs DC; △*P* < .05, △△*P* < .01 vs DG; □*P* < .05 vs DL; *P < .05 vs DGL

Co‐treatment with galanin and leptin altered bodyweight (*F*[7, 64] = 25.03, *P* < .0001) and food intake (*F*[7, 64] = 12.16, *P* < .001) of the rats. As shown in Figure 1, bodyweight and food intake in the both hormone group (DGL) reduced by 9.8% (*P* < .05) and 12.1% (*P* < .05) compared with the galanin group (DG), but increased by 13.8% (*P* < .05) and 16.6% (*P* < .05) compared with the leptin group (DL). In addition, bodyweight and food intake reduced by 12.3% (*P* < .05) and 13.6% (*P* < .05) in the galanin + leptin+MK‐2206 group (DGLM) compared with the galanin + leptin group (DGL), by 14.4% (*P* < .01) and 17.2% (*P* < .01) in the galanin + MK‐2206 group (DGM) compared with DG, and by 12.1% (*P* < .05) and 14.5% (*P* < .05) in the leptin group (DL) compared with the diabetic controls (DC). However, bodyweight and food intake increased by 13.8% (*P* < .05) and 14.9% (*P* < .05) in DGL compared with DL, by 12.9% (*P* < .05) and 14.9% (*P* < .05) in the leptin + MK‐2206 group (DLM) compared with DL, and by 10.9% (*P* < .05) and 13.4% (*P* < .05) in DG compared with DC. The bodyweight was lower, and the food intake was higher in DC than in the healthy controls (HC). Differences in the both indexes were non‐significant between DGLM and DGM, and between DGLM and DLM (*P *> .05).

### 3.2 HOMA index

Co‐treatment with galanin and leptin decreased HOMA index (*F*[7, 64] = 68.79, *P* < .0001) in the fat cells of the rats. The blood glucose and insulin levels, and HOMA index in the galanin + leptin group significantly attenuated by 11.2% (*P* < .05), 17.4% (*P* < .01) and 34.2% (*P* < .01) compared with the galanin group, and by 35.5% (*P* < .01), 20.7% (*P* < .05) and 48.6% (*P* < .01) compared with the leptin group, as shown in Table 1. Compared with the diabetic controls, the blood glucose and insulin levels, and HOMA index significantly reduced by 29.9% (*P* < .01), 22.1% (*P* < .01) and 45.4% (*P* < .01) in the galanin group, and by 14.3% (*P* < .05), 18.7% (*P* < .01) and 29.2% (*P* < .01) in the leptin group. In addition, the glucose and insulin levels, and HOMA index increased by 56.8% (*P* < .01), 64.1% (*P* < .01) and 153.5% (*P* < .01) in the galanin + leptin + MK‐2206 group than the galanin + leptin group, by 28.2% (*P* < .01), 23.9% (*P* < .01) and 57.7% (*P* < .01) in the galanin + MK‐2206 group compared with the galanin group, by 15.4% (*P* < .05), 33.8% (*P* < .01) and 53.4% (*P* < .01) in the leptin + MK‐2206 group compared with the leptin group. The three indexes were higher in the diabetic control group than in the healthy controls.

**TABLE 1:** The effects of central injection with galanin and leptin on HOMA indexes in rats (n = 8)

	Blood glucose (mmol/L)	Blood insulin (mmol/L)	HOMA indexes
HC	5.44 ± 0.53	4.25 ± 0.69	1.019 ± 0.12
DC	12.29 ± 1.16^○○^	6.58 ± 0.84^○○^	3.59 ± 0.41^○○^
Galanin	8.62 ± 0.68^●●^	5.13 ± 0.72^●●^	1.96 ± 0.18^●●^
Leptin	10.53 ± 1.03^●^	5.35 ± 0.65^●●^	2.51 ± 0.21^●●^
Gal + Lep	6.79 ± 0.53^△□□^	4.24 ± 0.62^△□^	1.29 ± 0.14^△△□□^
Gal + MK	11.05 ± 1.09^△△^	6.36 ± 0.76^△△^	3.09 ± 0.42^△△^
Lep + MK	12.15 ± 1.25^□^	7.16 ± 0.89^□□^	3.85 ± 0.45^□□^
Gal + Lep+MK	10.66 ± 0.97^**^	6.94 ± 0.77^**^	2.68 ± 0.28^**^
*F*[7, 64]	52.84	39.77	68.79
*P*	<.0001	<.0001	<.0001

Note

^○○^ *P* < .01 vs healthy control (HC) of each division; ^●^ *P* < .05, ^●●^ *P* < .01 vs diabetic control (DC) of each division; ^△^ *P* < .05, ^△△^ *P* < .01 vs galanin (Gal) group of each division; ^□^ *P* < .05, ^□□^ *P* < .01 vs leptin (Lep) group of each division, ^**^ *P* < .01 vs Gal + Lep group of each division. Mk‐2206 (MK).

### 3.3 Hyperinsulinaemic‐euglycaemic clamping test and [^3^H]2‐DG uptake

**FIGURE 2:**
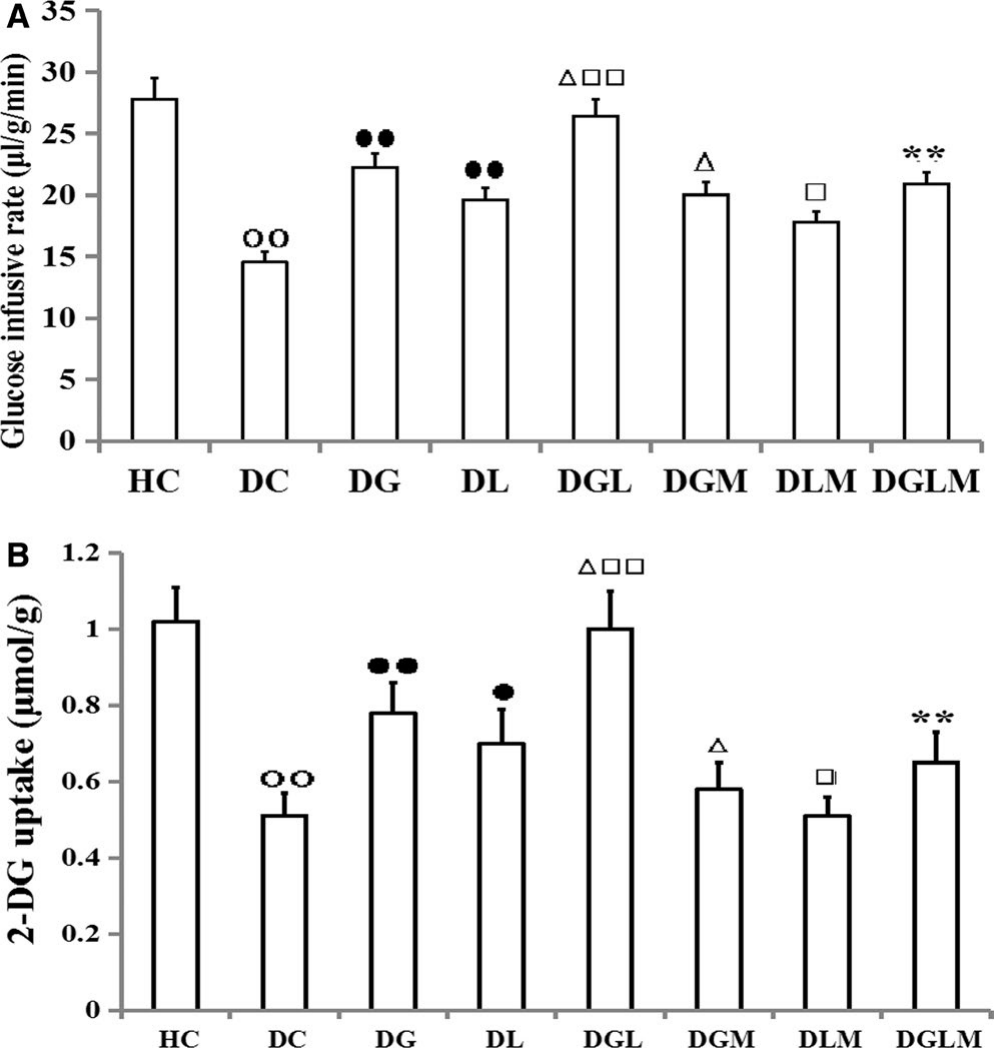
The effects of galanin and leptin on glucose infusing rates in hyperinsulinaemic‐euglycaemic clamp tests and glucose uptake in rats (n = 8). The glucose infusing rates and 2DG uptake were higher in the galanin + leptin group (DGL) than either galanin group (DG) or leptin group (DL), in DG or DL than diabetic controls (DC). However, the 2DG uptake and glucose infusing rates were lower in the both hormones + MK‐2206 group (DGLM) than DGL, in the galanin + MK‐2206 group (DGM) than DG, in DLM than DL and in DC than healthy controls (HC). Difference in both indexes was non‐significant between DGLM and DGM, and between DGLM and DLM. The data shown are the means ± SEM. ○○*P* < .01 vs HC; ●*P* < .05, ●●*P* < .01 vs DC; △*P* < .05, △△*P* < .01 vs DG; □*P* < .05, □□*P* < .01 vs DL; **P < .01 vs DGL

Co‐treatment with galanin and leptin increased glucose infusion rates (*F*[7, 64] = 26.81, *P* < .0001) in the hyperinsulinaemic‐euglycaemic clamp tests (Figure 2A) and [^3^H]2‐DG uptake (*F*[7, 64] = 26.81, *P* < .0001) in the fat cells of the rats (Figure 2B). During the clamp tests, glucose infusion rates and [^3^H]2‐DG levels enhanced by 18.8% (*P* < .05) and 24.6% (*P* < .05) in DGL compared with DG, and 34.5% (*P* < .01) and 38.9% (*P* < .01) in DGL compared with DL, and by 53.1% (*P* < .01) and 51.4% (*P* < .01) in DG compared with DC, and 35.1% (*P* < .01) and 35.8% (*P* < .05) in DL compared with DC, respectively. However, infusion rates and [^3^H]2‐DG uptake attenuated by 25.6% (*P* < .01) and 32.6% (*P* < .01) in DGLM compared with DGL, and by 10.1% (*P* < .05) and 24.9% (*P* < .05) in DGM compared with DG, and by 9.4% (*P* < .05) and 26.4% (*P* < .05) in DLM compared with DL, respectively. In addition, infusion rates and [^3^H]2‐DG levels in DC were higher than in HC (*P* < .01). Differences in the both indexes were non‐significant between DGLM and DGM, as well as DGLM and DLM (*P *> .05), respectively.

### 3.4 GLUT4 expression in fat cells

**FIGURE 3:**
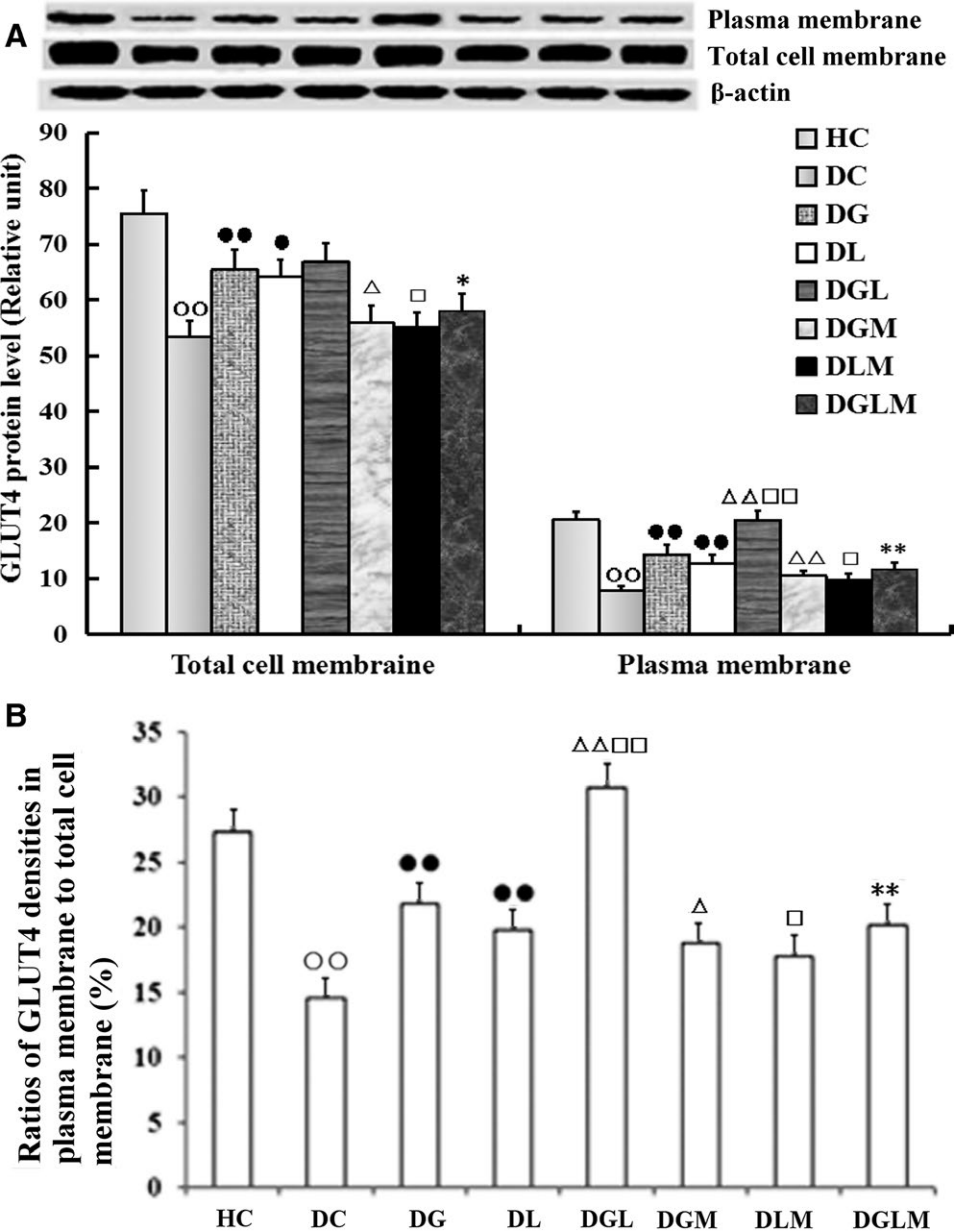
The central effect of galanin and leptin on GLUT4 trafficking to plasma membranes of fat cells (n = 8). The GLUT4 levels in plasma membranes were higher in the galanin + leptin group (DGL) than either galanin group (DG) or leptin group (DL), but not in total cell membranes (A). The ratios of the former to the latter were increased too (B). The GLUT4 levels in both membranes and their ratios were higher in DG or DL than diabetic controls (DC), but lower in the both hormones + MK‐2206 group (DGLM) than DGL, in the galanin + MK‐2206 group (DGM) than DG, in DLM than DL, and in DC than healthy controls (HC). Difference in the levels in both membranes and their ratio was non‐significant between DGLM and DGM, and between DGLM and DLM. The data shown are the means ± SEM. ○○*P* < .01 vs HC; ●*P* < .05, ●●*P* < .01 vs DC; △*P* < .05, △△*P* < .01 vs DG; □*P* < .05, □□*P* < .01 vs DL; *P < .05, **P < .01 vs DGL

Co‐treatment with galanin and leptin increased GLUT4 expression in the total cell membranes (*F*[7, 64] = 14.78, *P* < .001) and the plasma membranes (*F*[7, 64] = 51.82, *P* < .0001), as well as the ratios of GLUT4 levels in the plasma membranes to that in the total cell membranes (*F*[7, 64] = 129.46, *P* < .0001) in the fat cells of the rats. As compared with injection of galanin or leptin alone, GLUT4 levels in DGL elevated by 43.7% (*P* < .01) and 70.2% (*P* < .01) in the plasma membranes, whereas by only 2.1% (*P *> .05) and 4.2% (*P *> .05) in the total cell membranes (Figure 3A), and the ratios of GLUT4 densities in the plasma membranes to the total cell membranes increased by 40.6% (*P* < .01) and 54.9% (*P* < .01), respectively (Figure 3B). Likewise, in comparison with DC, the GLUT4 immunoreactivities elevated by 83.5% (*P* < .01) in the plasma membranes of DG and by 54.9% (*P* < .01) of DL, by 22.7% (*P* < .01) in the total cell membranes of DG and by 20.2% (*P* < .01) of DL, and by 49.6% (*P* < .01) in their ratios (*P* < .01) of DG and by 35.8% (*P* < .01) of DL, respectively. However, the GLUT4 contents in plasma membranes, total cell membranes and their ratios reduced by 43.1% (*P* < .01), 13.1% (*P* < .05) and 34.4% (*P* < .01) in DGLM compared with DGL, 6.2% (*P* < .01), 14.7% (*P* < .05) and 13.8% (*P* < .05) in DGM compared with DG, and by 22.9% (*P* < .05), 13.1% (*P* < .05) and 10.3% (*P* < .05) in DLM compared with DL. The three parameters were significantly lowered in DC than in HC (*P* < .01). Differences in the immunoreactivities in both membranes and their ratios were non‐significant between DGLM and DGM, and between DGLM and DLM (*P *> .05).

### 3.5 GLUT4 mRNA expression in fat cells and plasma adiponectin levels

**FIGURE 4:**
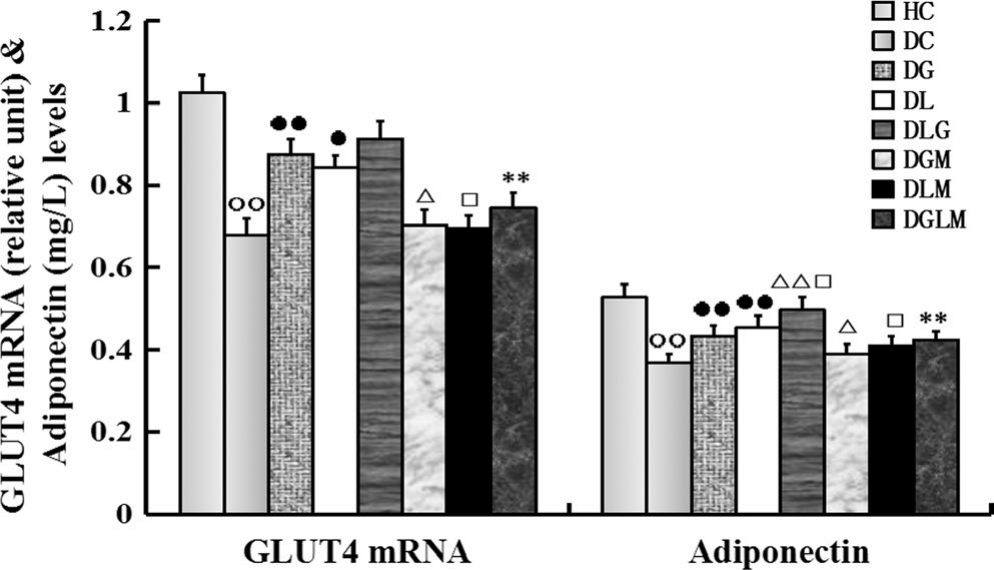
The central effect of galanin and leptin on GLUT4 mRNA expression in adipose cells and plasma adiponectin levels in rats (n = 8). The plasma adiponectin levels were higher in the galanin + leptin group (DGL) than either galanin group (DG) or leptin group (DL), but GLUT4 mRNA expression in fat cells was almost changeless in DGL compared with DG and DL, respectively. Both indexes were higher in DG or DL than diabetic controls (DC), whereas that were lower in the both hormones + MK‐2206 group (DGLM) than DGL, in the galanin + MK‐2206 group (DGM) than DG, in DLM than DL and in DC than healthy controls (HC). Differences in the both indexes were non‐significant between DGLM and DGM, and between DGLM and DLM. The data shown are the means ± SEM. ○○*P* < .01 vs HC; ●*P* < .05, ●●*P* < .01 vs DC; △*P* < .05, △△*P* < .01 vs DG; □*P* < .05 vs DL; **P < .01 vs DGL

Co‐treatment with galanin and leptin increased GLUT4 mRNA expression (*F*[7, 64] = 14.94, *P* < .001) in the fat cells and plasma adiponectin levels (*F*[7, 64] = 38.34, *P* < .0001) of the rats. The plasma adiponectin levels increased by 14.5% (*P* < .01) and 9.5% (*P* < .05), whereas GLUT4 mRNA expression in epididymal adipose tissue increased only by 2.1% (*P *> .05) and 4.3% (*P *> .05) in DGL compared with DG and DL, respectively (Figure 4). Both adiponectin and GLUT4 mRNA levels decreased by 18.3% (*P* < .01) and 14.7% (*P* < .01) in DGLM compared with DGL, by 19.6% (*P* < .01) and 10.2% (*P* < .05) in DGM compared with DG, and by 17.5% (*P* < .05) and 9.5% (*P* < .05) in DLM compared with DL, respectively. However, adiponectin and GLUT4 mRNA levels increased by 17.3% (*P* < .01) and 28.9% (*P* < .01) in DG compared with DC, by 22.8% (*P* < .01) and 24.9% (*P* < .05) in DL compared with DC, respectively. Both levels in DC were lower than in HC (*P* < .01). Differences in the both indexes were non‐significant between DGLM and DGM, and between DGLM and DLM (*P *> .05).

### 3.6 Akt phosphorylation

**FIGURE 5:**
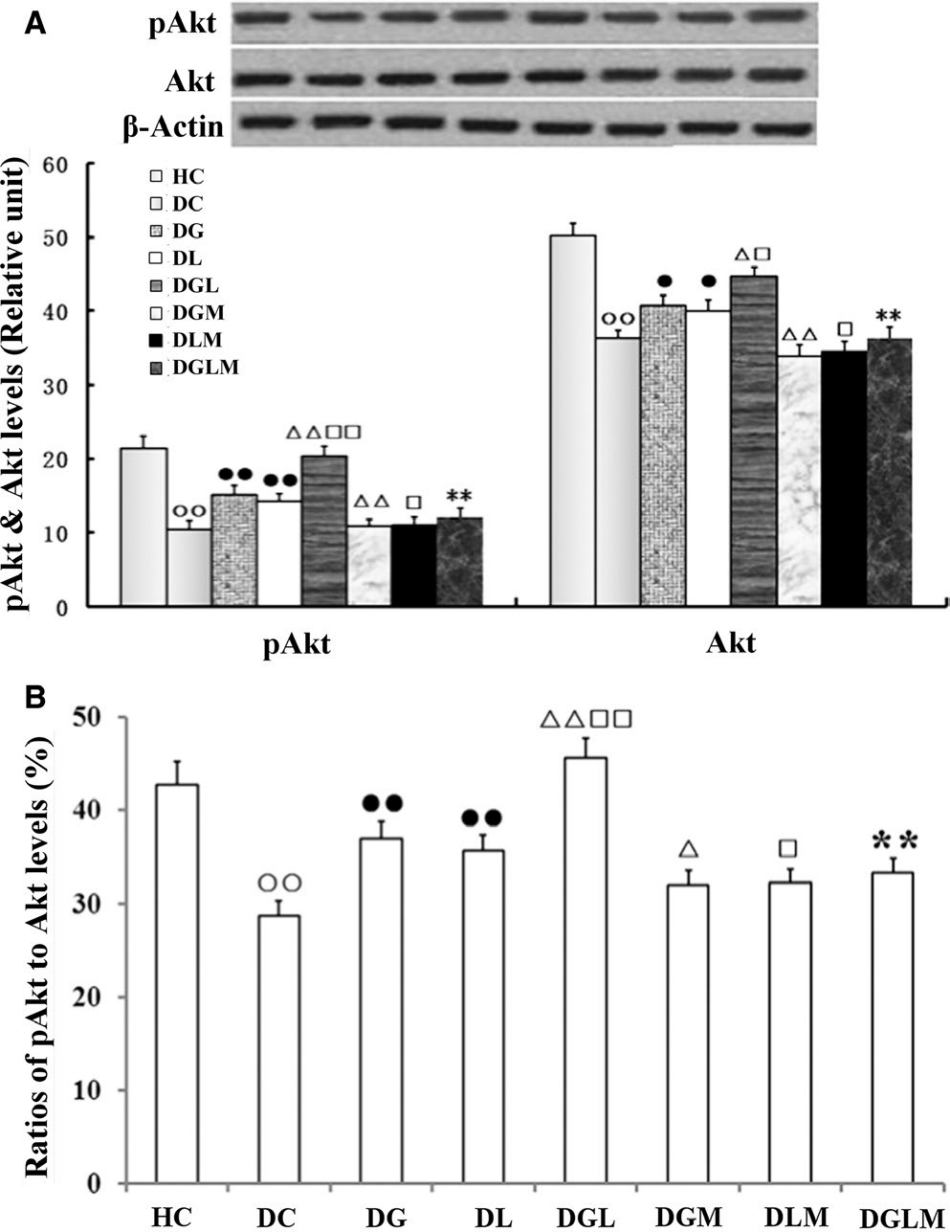
The central effect of galanin and leptin on pAkt and Akt levels and pAkt/Akt ratios in the fat cells of rats (n = 8). pAkt and Akt levels and their ratio were higher in the galanin + leptin group (DGL) than either galanin group (DG) or leptin group (DL), in DG or DL than diabetic controls (DC). However, the three indexes were lower in the both hormones + MK‐2206 group (DGLM) than DGL, in the galanin + MK‐2206 group (DGM) than DG, in DLM than DL and in DC than healthy controls (HC). Difference in the three indexes was non‐significant between DGLM and DGM, and between DGLM and DLM. The data shown are the means ± SEM. ○○*P* < .01 vs HC; ●*P* < .05, ●●*P* < .01 vs DC; △*P* < .05, △△*P* < .01 vs DG; □*P* < .05, □□*P* < .01 vs DL; **P < .01 vs DGL

Co‐treatment with galanin and leptin increased pAkt (*F*[7, 64] = 54.45, *P* < .001) and Akt (*F*[7, 64] = 50.72, *P* < .001) expression in the fat cells and the ratios of pAkt/Akt (*F*[7, 64] = 121.89, *P* < .0001) of the rats. As shown in Figure 5A,B, pAkt and Akt contents, as well as their ratios, significantly elevated by 35.1% (*P* < .01), 9.5% (*P* < .05) and 28.6% (*P* < .01) in DGL compared with DG, and 43.1% (*P* < .01), 11.8% (*P* < .05) and 24.1% (*P* < .01) in DGL compared with DL, but attenuated by 40.6% (*P* < .01), 18.5% (*P* < .05) and 27.1% (*P* < .01) in DGLM compared with DGL, by 24.5% (*P* < .01), 16.7% (*P* < .01) and 13.4% (*P* < .05) in DGM compared with DG, and by 19.9% (*P* < .05), 13.6% (*P* < .05) and 9.5% (*P* < .05) in DLM compared with DL, respectively. Compared with DC, the pAkt and Akt contents, as well as their ratios, enhanced by 43.5% (*P* < .01), 12.2% (*P* < .05) and 28.6% (*P* < .01) in DG, by 36.2% (*P* < .01), 9.9% (*P* < .05) and 24.1% (*P* < .01) in DL, respectively. The three parameters in DC were lower than HC (*P* < .01). Differences in the three indexes were non‐significant between DGLM and DGM, and between DGLM and DLM (*P*> .05).

## 4 DISCUSSION

The purpose of current study was to investigate the combined effect of central leptin and galanin on insulin resistance in adipose cells. We found that the actions of leptin and galanin are both opposite and cooperative. On the one hand, the effects of leptin and galanin on energy metabolism are conflictive. Administration of leptin increased energy expenditure, but reduced food intake and bodyweight, whereas injection with galanin decreased energy expenditure and stimulated food intake and weight gain in animals.^7^, ^15^ Activation of galanin receptor subtype 2 in the lateral hypothalamus reinstated blunted leptin sensitivity during hedonic overeating of palatable foods in lean animals.^24^ The results in the present study showed that administration of both hormones compromised their effects on food intake and bodyweight. The combined roles of both hormones were lower than galanin, but higher than leptin. On the other hand, the effects of galanin and leptin on insulin sensitivity are cooperative. Bektur et al found that an antidepressant, mirtazapine, showed an anti‐hyperglycaemic effect by decreasing galanin receptor subtype 2 through altering the leptin and galanin expression in the liver of type 1 diabetic rats.^25^

Aforementioned, administration of either leptin or galanin may ameliorate insulin resistance in the diabetic animals. To assess the impacts of both hormones on insulin resistance, we surveyed several indexes of insulin sensitivity in the current study. The first is [^3^H]2‐DG uptake. After transported into cells, [^3^H]2‐DG cannot be metabolized for glycolysis as glucose does. So, the quantity of trapped [^3^H]2‐DG in cells becomes a biomarker of glucose uptake and insulin sensitivity.^26^ The second is the glucose infusion rates in hyperinsulinaemic‐euglycaemic clamp tests, which is a classical measurement of insulin sensitivity.^12^ The third is GLUT4 translocation from intracellular storage organelles onto plasma membranes. It is acknowledged that only after transported onto the cell surface, GLUT4 can move glucose into cells. So, the GLUT4 levels in plasma membranes of cells reflect glucose clearance and insulin sensitivity.^9^, ^27^ The fourth is the plasma adiponectin concentration. Secreted by adipose tissues, adiponectin shows anti‐inflammatory and anti‐diabetic properties.^28^ Adiponectin‐deficient mice exhibited insulin resistance and diabetic symptom.^29^ Conversely, injection of recombinant adiponectin or overexpression of adiponectin prevented development of diabetes and hyperlipidaemia.^28^, ^30^ The last is HOMA index. By means of a simple mathematically derived equation, HOMA index offers an indirect assessment of insulin sensitivity in clinic and laboratory through the measurement of fasting glucose and insulin levels. The results of the present study revealed that i.c.v. treatment with either leptin or galanin improved these parameters of insulin resistance, and co‐administration of both hormones further optimized them and enhanced insulin sensitivity in diabetic animals.

Noteworthily, the GLUT4 mRNA level more likely reflects the change in GLUT4 synthesis rate rather than its half‐life.^11^ In this experiment, GLUT4 mRNA and total GLUT4 protein expression levels were almost changeless in the galanin + leptin group compared with leptin‐ or galanin‐treated group, suggesting that co‐administration of both hormones was unable further to enhance the GLUT4 levels in comparison with anyone did in the fat cells.

There is an interaction between leptin and galanin in their synthesis and secretion. Treatment with galanin reduced leptin expression and secretion in a dose‐dependent manner in the adipose tissue of fasting rats.^31^ Accordingly, galanin knockout mice showed increased circulating leptin levels and leptin sensitivity to reduce their bodyweight and fat pad mass,^32^ that is deletion of galanin gene amplified leptin‐induced weight loss.^33^ In turn, leptin significantly increased the galanin serum level and gene expression in the hypothalamus.^33^, ^34^ Thus, there is a negative feedback in their synthesis between leptin and galanin, the former to the latter is promoting, and the latter to the former is inhibitive. This negative feedback is helpful to maintain the homeosta of both hormones to keep energy homeostasis.

In order to understand the mechanism of cooperation between galanin and leptin in amelioration of insulin resistance, we surveyed Akt phosphorylation, a key link of signalling systems of both hormones.^35^, ^36^ Recent studies showed that leptin receptor mediated the regulation of the glucose metabolism via activation of AKT pathway.^37^ The AKT inhibitor can significantly counteracted the effect of leptin, and AKT activator can counteracted the effect of leptin silencing. In addition, galanin can benefit glucose uptake via inhibition of adenylyl cyclase and cAMP through Gi/o receptors to activate the PI3K/Akt signalling pathway too.^38^ Thus, Akt becomes a meeting point of both signalling pathways to regulate glucose uptake and metabolism. This overlap may result in enhancing the signal intensity and transmitting efficacy to increase insulin sensitivity. Yuan et al found that galanin might modulate the leptin signals, and the inhibiting effects on neuron activity were stronger after co‐application of galanin and leptin into the gastric compartment than application of galanin or leptin alone in the nucleus tractus solitarius.^39^ In the current study, combination of both hormones stimulated more Akt phosphorylation than either leptin or galanin alone did, which may be blocked by MK‐2206, suggesting that Akt was necessary for the cooperative effects of both hormones on alleviation of insulin resistance.

In summary, the results of our present study showed that the central effect of galanin and leptin on appetite and bodyweight of animals is opposite, but that on amelioration of insulin resistance is cooperative. The co‐administration of both hormones further enhanced glucose infusion rates in the clamp tests, plasma adiponectin content, 2‐deoxy‐D‐[^3^H]glucose uptake, GLUT4 translocation and Akt phosphorylation in the adipose tissues, but reduced HOMA index, did not affect GLUT4 mRNA and total GLUT4 expression levels. Besides, the Akt phosphorylation is necessary for the cooperative effects of both hormones in the fat cells. These findings deepen our understanding of the interrelation between galanin and leptin in mitigation of insulin resistance, and provide an experimental clue for further testing whether co‐administration of both hormones may get better efficacy against insulin resistance than treatment with galanin or leptin alone for type 2 diabetic patients.

## CONFLICT OF INTEREST

The authors have no conflicts of interest to disclose.

## AUTHOR CONTRIBUTIONS

LB and SQ contributed to the design and writing of the manuscript. L.B and XC were involved in conduct/data collection and critical analysis.

## Data Availability

The data that support the findings of this study are available from the corresponding author upon reasonable request.
